# Dietary *n*-6:*n*-3 PUFA Ratio Modulates Inflammation-Related Gene Expression and Influences Improvements in Biochemical Parameters in a Murine Model of Diet-Induced Obesity

**DOI:** 10.3390/nu17121996

**Published:** 2025-06-13

**Authors:** Alejandro Gutierrez-Guerra, Diego Cambron-Mora, Roberto Rodriguez-Echevarria, Jorge Hernández-Bello, Wendy Campos-Pérez, Alejandro A. Canales-Aguirre, Mariana Pérez-Robles, Erika Martinez-Lopez

**Affiliations:** 1 Instituto de Nutrigenética y Nutrigenómica Traslacional, Departamento de Biología Molecular y Genómica, Centro Universitario de Ciencias de la Salud, Universidad de Guadalajara, Guadalajara 44340, Mexico; alegtzguerra@gmail.com (A.G.-G.); diego.cambron@alumnos.udg.mx (D.C.-M.); juan.rodriguez8035@academicos.udg.mx (R.R.-E.); wendy.campos4381@academicos.udg.mx (W.C.-P.); mariana.perez@academicos.udg.mx (M.P.-R.); 2 Doctorado en Ciencias Biomédicas, Centro Universitario de Ciencias de la Salud, Universidad de Guadalajara, Guadalajara 44340, Mexico; 3 Doctorado en Ciencias en Biologia Molecular en Medicina, Centro Universitario de Ciencias de la Salud, Universidad de Guadalajara, Guadalajara 44340, Mexico; 4 Instituto de Investigación en Ciencias Biomédicas, Departamento de Clínicas Médicas, Centro Universitario de Ciencias de la Salud, Universidad de Guadalajara, Guadalajara 44340, Mexico; jorge.hernandezbello@cucs.udg.mx; 5 Unidad de Evaluación Preclínica, Unidad de Biotecnología Médica y Farmacéutica, Centro de Investigación y Asistencia en Tecnología y Diseño del Estado de Jalisco, CIATEJ, Guadalajara 44270, Mexico; acanales@ciatej.mx

**Keywords:** *n*-6:*n*-3 ratio, diet-induced obesity, murine model, biochemical parameters, inflammatory cytokines, antioxidant genes

## Abstract

**Background**: An unbalanced dietary intake of omega-6 (*n*-6) and omega-3 (*n*-3) polyunsaturated fatty acids (PUFAs) has been associated with chronic inflammation and oxidative stress, both of which contribute to the pathophysiology of obesity. **Objective**: We aimed to evaluate the effects of a diet with an *n*-6:*n*-3 PUFA ratio of 5:1 on body composition, biochemical parameters, and the gene expression of cytokines and antioxidant enzymes in a murine model of diet-induced obesity. **Methods**: A diet-induced obesity model was established in C57BL6/J mice over 17 weeks. Mice were then fed different diets for 8 weeks: a control diet (chow), a high-fat diet with a 30:1 *n*-6:*n*-3 ratio (HFD-30:1), and a high-fat diet enriched with *n*-3 fatty acids, with a 5:1 *n*-6:*n*-3 ratio (HFD-5:1). Body weight and food intake were monitored throughout this study. Biochemical parameters were measured, and the expression of antioxidant enzymes and cytokine genes was analyzed by qPCR. Data were analyzed using GraphPad Prism software. **Results**: The HFD-5:1 group exhibited a significant reduction in body weight (*p* = 0.0182), liver tissue weight (*p* = 0.01), serum glucose levels (*p* = 0.010), area under the curve (AUC) (*p* = 0.0161), cholesterol (*p* < 0.0001), and triglycerides (*p* = 0.0069) compared to the HFD-30:1 group. The body weight in the HFD-5:1 group decreased to levels comparable to the control group. Additionally, the expression of the inflammatory cytokine genes *Ccl2* (*p* = 0.0389) and *Tgfb1* (*p* = 0.0226) was significantly reduced. **Conclusions**: These findings suggest that adjusting the dietary *n*-6:*n*-3 ratio to 5:1 modulates inflammation-related gene expression and improves metabolic markers in obese mice, supporting its potential relevance for future translational research.

## 1. Introduction

Obesity is defined by the World Health Organization (WHO) as an abnormal or excessive accumulation of fat, typically identified by a body mass index (BMI) of 30 kg/m^2^ or greater [1,2]. In 2022, it was reported that 890 million people worldwide were living with obesity, equating to one in eight individuals [3]. Obesity is a major public health problem, as the accumulation of intra-abdominal and intravascular fat is linked to serious comorbidities, including type 2 diabetes mellitus (T2DM), hypertension, coronary heart disease, hyperlipidemia, and an increased risk of certain cancer types, such as colon, prostate, endometrial, and breast cancer [4].

Obesity is characterized by chronic low-grade inflammation, with elevated serum levels of pro-inflammatory cytokines [5]. It is also associated with oxidative stress, marked by an imbalance between the amount of reactive oxygen species (ROS) and the antioxidant defenses that neutralize them [6]. These processes are closely interconnected, as inflammatory mediators can induce oxidative stress, creating a self-perpetuating feedback loop [7].

One strategy to address obesity involves dietary interventions aimed at modifying food intake patterns and adjusting calories and macronutrient composition, particularly the types of fat consumed [8]. Polyunsaturated fatty acids (PUFAs) are essential nutrients that must be obtained from the diet [9]. They are classified into two main families: omega-6 (*n*-6) and omega-3 (*n*-3). A higher proportion of *n*-6 intake has been associated with pro-inflammatory effects, potentially contributing to the development of obesity, atherosclerosis, and T2DM. In contrast, *n*-3 fatty acids exhibit regulatory activity in hepatic lipogenesis, as well as anti-inflammatory and antithrombotic properties. A beneficial *n*-6:*n*-3 ratio has been proposed in a range from 1:1 to 5:1 [10,11].

The beneficial effects of a higher intake of *n*-3 fatty acids, especially when the *n*-6:*n*-3 ratio approaches a balance, are linked to the antioxidant activities of *n*-3 derivatives. Specifically, eicosapentaenoic acid (EPA) and docosahexaenoic acid (DHA) have been shown to exert antioxidant effects via the Nrf2 pathway in adipocytes. This pathway plays a critical role in cellular defense mechanisms by regulating the expression of antioxidant proteins that protect against oxidative damage induced by inflammatory processes and metabolic stress [12].

Some studies suggest that a lower *n*-6:*n*-3 ratio may improve metabolic parameters in adolescents with obesity and fatty liver disease [13], enhance glucose and lipid metabolism in T2DM [14], and decrease serum levels of TNF-α, IL-1β, IL-6, and MCP-1 [15].

Achieving an optimal *n*-6:*n*-3 ratio remains challenging due to modern dietary patterns, particularly the widespread consumption of processed foods and cooking oils rich in *n*-6 fatty acids. This imbalance is compounded by the limited intake of *n*-3-rich sources such as fish, flaxseeds, and nuts [11,16,17]. Nonetheless, several studies have demonstrated that dietary interventions aiming to achieve a 5:1 *n*-6:*n*-3 ratio can attenuate the adverse metabolic and inflammatory effects associated with an excessive intake of *n*-6 fatty acids [18]. This raises the following research question: Can a dietary *n*-6:*n*-3 PUFA ratio of 5:1 improve metabolic parameters and modulate the expression of genes related to inflammation and antioxidant activity in a murine model of diet-induced obesity? Therefore, the aim of this study was to evaluate the impact of a diet with an *n*-6:*n*-3 PUFA ratio of 5:1 on body composition, biochemical parameters, cytokines levels, and antioxidant enzyme expression in a murine model of diet-induced obesity compared with a diet with an *n*-6:*n*-3 PUFA ratio of 30:1.

We hypothesized that this dietary ratio would improve metabolic profiles, accompanied by reduced inflammation and oxidative stress through the regulation of key molecular pathways. To our knowledge, few studies have assessed these specific ratios in an obesity model focusing on gene expression in adipose tissue.

## 2. Materials and Methods

### 2.1. Animals and Groups

For this study, male C57BL/6J mice, aged 5–6 weeks and weighing 20–25 g, were obtained from the Institute of Neurobiology at the Universidad Autónoma de México (Mexico City, Mexico). The mice were housed in groups of five per cage in a temperature- and humidity-controlled room, under a 12 h light/dark cycle, with access to standard chow and water ad libitum. After one week of acclimatization, the animals were randomly assigned to two equal groups. The first group (*n* = 10, control) was fed a standard diet (D12450H, Research Diets, New Brunswick, NJ, USA) consisting of 10% lipids, 70% carbohydrates, and 20% protein. The second group (HFD 30:1, *n* = 15) received a customized high-fat diet (D21022504, Research Diets, based on the D12451 formula), which contained 45% lipids, 35% carbohydrates, and 20% protein. The HFD 30:1 diet represents a Westernized dietary pattern, characterized by a high *n*-6 to *n*-3 fatty acid ratio of 30:1. After 17 weeks of feeding, five mice from each group were sacrificed to collect blood and adipose tissue samples for model validation. The remaining HFD-fed mice were subdivided into two groups: one continued on an HFD-30:1 diet, while the other was switched to the HFD-5:1 formulation. Mice in the control group continued on the control diet for an additional 8 weeks. Euthanasia was performed using a carbon dioxide chamber with a flow rate of 20% of the chamber volume per min until a CO_2_ concentration of 70% was reached, ensuring rapid loss of consciousness.

### 2.2. Diets

The HFD-5:1 (D21022505) and the HFD-30:1 (D21022504) diets were formulated by the research team to optimize macronutrient distribution and ensure a precise *n*-3 fatty acid content in each formulation. These two high-fat diets with differing *n*-3 contents, along with the control diet (D12450H), were ordered from Research Diet, Inc. (New Brunswick, NJ, USA). Table 1 presents the ingredients, macronutrient distribution (kcal/g), and *n*-6:*n*-3 ratio of each diet.

### 2.3. Fasting Glucose and Insulin Tolerance Test (ITT)

Mice were fasted for 4 h before the procedure. A small distal incision was made in the tail to obtain blood samples. Glucose levels were measured at week 17 and at the end of the 8-week intervention using a glucometer (ACCU-CHEK Active, Roche, Mannheim, Germany). The insulin tolerance test (ITT) was performed at 0, 15, 30, 45, 60, 75, and 90 min following intraperitoneal injection of insulin (1 U/kg body weight) diluted in 0.9% saline solution. The area under the curve (AUC) was calculated using the trapezoidal method for each experimental group.

### 2.4. Serum Biochemistry Analysis

Plasma and collected tissues were immediately snap-frozen and stored at −80 °C until analysis. Blood samples were collected after a 4–6-h fasting period to measure triglycerides and total cholesterol using a standard clinical analyzer (Vitros 350, Ortho Clinical Diagnostics, Raritan, NJ, USA) according to the manufacturer’s protocol.

### 2.5. Analysis of Gene Expression by Quantitative Real-Time PCR

Adipose tissue samples were ground using a POLYTRON^®^ PT 2500 E (Kinematica, Malters, Switzerland) and subsequently homogenized in TRIzol^®^ reagent (Invitrogen, Carlsbad, CA, USA). Total RNA was isolated from 100 mg of cold-homogenized adipose tissue. After incubation on ice, chloroform was added, and the sample was mixed manually and vortexed. The aqueous phase was extracted and precipitated with isopropanol, followed by incubation and centrifugation. The sample was washed with 75% ethanol, the ethanol was removed, and the pellet was resuspended in 0.1% DEPC-treated water. The RNA concentration and purity were assessed using a MultiScan Sky spectrophotometer (Thermo Fisher Scientific^®^, Waltham, MA, USA). RNA integrity was evaluated in 1% agarose gel electrophoresis.

Complementary DNA (cDNA) was synthesized using 2 μL of total RNA (500 ng/μL). M-MLV reverse transcriptase (Invitrogen™, Carlsbad, CA, USA) was used under the following conditions: incubation at 25 °C for 10 min, followed by reverse transcription at 37 °C for 60 min. The enzyme was subsequently inactivated by heating the mixture to 95 °C for 10 min. Synthesized cDNA was stored at −80 °C until further use.

qPCR was performed using TaqMan probes (2×) according to the manufacturer’s instructions. Gene expression analysis included markers associated with inflammation (*Il1a*, *Il1b*, *Il4*, *Il6*, *Il10*, *Il13*, *Tnfa*, *Ccl2*, *Tgfb1*, *Ifng*) and with the antioxidant system (*Gpx1*, *Sod1*, *Cat*). The catalog numbers and Assay IDs of each TaqMan probe used are detailed in Supplementary Table S1.

All samples were run in duplicate and normalized to the housekeeping gene *Eif3f* (Cat. No. 4448490; Assay ID: Mm00517953_m1). Quantification of target gene expression was performed on a LightCycler^®^ 96 instrument (Roche, Mannheim, Germany) using the following cycling conditions: initial incubation at 50 °C for 2 min, followed by 40 cycles of 95 °C for 15 s and 60 °C for 60 s. The reaction mixture consisted of 1 μL of molecular biology-grade water, 2.5 μL of FastStart Essential DNA Probes Master Mix (TaqMan^®^ Universal PCR, 1×), 0.5 μL of TaqMan^®^ probe (2×), and 1 μL of cDNA. Relative gene expression levels were calculated using the 2^−∆Cq^ method [19].

### 2.6. Statistical Analysis

All data were assessed for normality using the Shapiro–Wilk test to determine the appropriate statistical tests for subsequent analyses. Comparisons between two independent experimental groups were performed using either the unpaired Student’s *t*-test (for normally distributed data) or the Mann–Whitney *U* test (for non-normally distributed data), to assess differences in central tendency. For comparisons involving more than two groups, one-way analysis of variance (ANOVA) was used when parametric assumptions were met, followed by Bonferroni’s post hoc test to assess pairwise differences. When data did not meet parametric assumptions, the Kruskal–Wallis test was applied as a non-parametric alternative, followed by Dunn’s post hoc test for multiple comparisons. To correct for multiple testing, a Bonferroni adjustment was applied for five comparisons, resulting in an adjusted significance threshold of *p* < 0.05. All statistical analyses were conducted using GraphPad Prism version 8. Data are presented as the mean ± standard deviation (SD), unless otherwise specified. A *p*-value < 0.05 was considered statistically significant.

## 3. Results

### 3.1. Impact of n-6:n-3 Fatty Acid Ratios on Body Weight Progression, Tissue Weights, Cytokine Expression, and Antioxidant Enzyme Profiles

From the first week of the experiment, animals were divided into two groups and fed either a control diet or a high-fat diet (HFD). Body weight was monitored in both groups over 17-week period. At the end of the obesity induction period, HFD-fed animals exhibited a 27% increase in body weight compared to the control group (*p* < 0.0001; Figure S1). Additionally, increased weights of epididymal adipose tissue (*p* = 0.001) and liver (*p* = 0.005) and an increased relative liver weight (*p* = 0.039) were observed (Table S2), confirming the successful establishment of the obesity model. In this model, the HFD group exhibited significantly higher levels of triglycerides (Figure S2A), cholesterol (Figure S2B), and glucose (Figure S2C) compared to the control group (*p* = 0.0104).

Cytokine expression profiling in adipose tissue revealed elevated mRNA levels of *Il1a*, *Il1b*, *Il4*, *Il6*, *Il10*, *Tgfb1*, and *Il13* in the HDF-30:1 group compared to the control group (Figure S3A–E,H,I). Although expression levels of *Tnfa* and *Ccl2* were evaluated in HFD-30:1 mice, the differences did not reach statistical significance (Figure S3F,G). No significant differences were observed in the expression of antioxidant-related genes (*Gpx1*, *Sod1*, and *Cat*) (Figure S4A–C).

### 3.2. Effect of the 8-Week Dietary Intervention with HFD-5:1

#### 3.2.1. Body Weight and Caloric Intake

**Figure 1:**
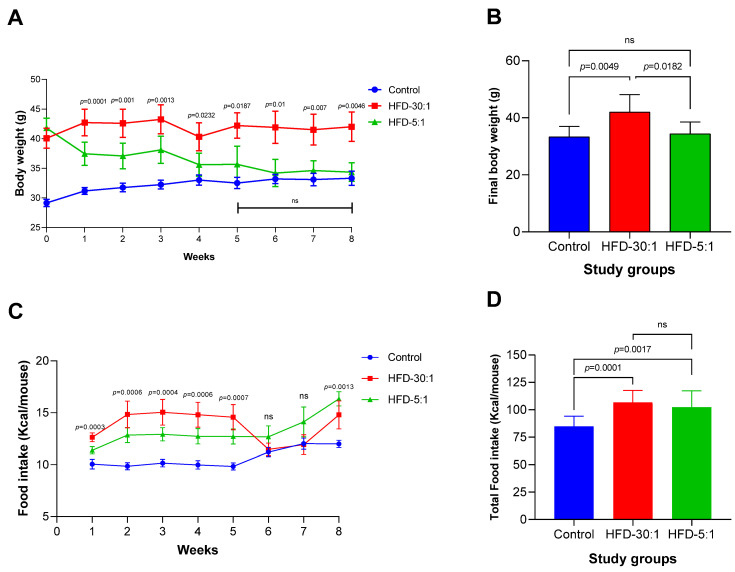
Body weight and energy intake during and after the 8-week dietary intervention. (**A**) Body weight progression over time, where week 0 represents baseline values; (**B**) final body weight at the end of the intervention; (**C**) weekly food intake per mouse; (**D**) total caloric intake per mouse over the 8-week period. Data are presented as mean ± SD. Statistical differences were assessed using one-way ANOVA followed by post hoc comparisons. ns: non-significant.

After confirming the establishment of the obesity model, body weight was monitored throughout the 8-week intervention period in the three study groups (Figure 1A). At the end of the 8-week treatment, a significant reduction in body weight was observed in the HFD-5:1 group compared to the HFD-30:1 group; body weight in the HFD-5:1 group decreased to levels similar to those of the control group (Figure 1B). To assess whether these results were influenced by caloric intake, food consumption was evaluated. However, no significant differences in food intake were observed between the two HFD groups (Figure 1C,D).

Upon completion of the 8-week intervention, no significant differences were observed in epididymal adipose tissue among the groups (*p* = 0.4). However, consistent with the reduction in body weight (Figure 1B), the group receiving the 5:1 *n*-6:*n*-3 PUFA intervention exhibited a significant decrease in both absolute and relative liver weights (Table 2).

#### 3.2.2. Insulin Tolerance Test (ITT)

**Figure 2:**
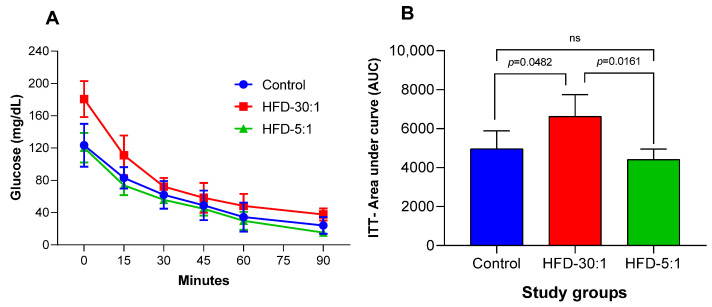
Insulin tolerance test (ITT) and glucose area under the curve (AUC) following the 8-week dietary intervention. (**A**) Glucose levels over time during the ITT (0–90 min); (**B**) glucose AUC calculated from ITT measurements. Data are presented as mean ± SD. Statistical comparisons were performed using one-way ANOVA followed by post hoc analysis. ns: non-significant.

The ITT was conducted to calculate the area under the curve (AUC) for glucose, with samples collected every 15 min over a 90 min period (at 0, 15, 30, 45, 60, and 90 min). A significant reduction in glucose AUC was observed in the HDF-5:1 group, reaching levels lower than those of the control group, as shown in Figure 2A,B.

#### 3.2.3. Biochemical Parameters

**Figure 3:**
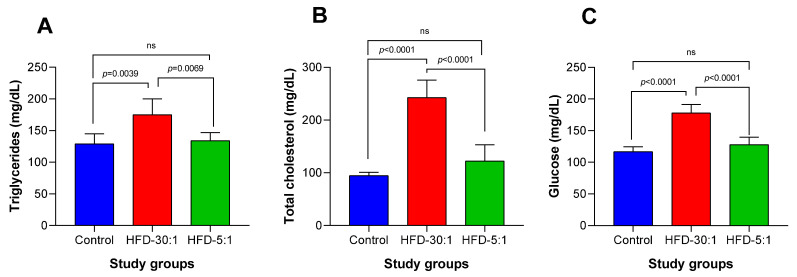
Levels of triglycerides, total cholesterol, and glucose at the end of the 8-week dietary intervention. (**A**) Triglyceride levels; (**B**) total cholesterol levels; (**C**) glucose levels. Values are presented as mean ± SD. ns: non-significant.

Biochemical parameters (glucose, triglycerides, and total cholesterol) were significantly reduced in the HDF-5:1 group compared to the HFD-30:1 group (Figure 3). No significant differences were observed between the HFD-5:1 and control groups.

#### 3.2.4. Cytokine Expression Profile

**Figure 4:**
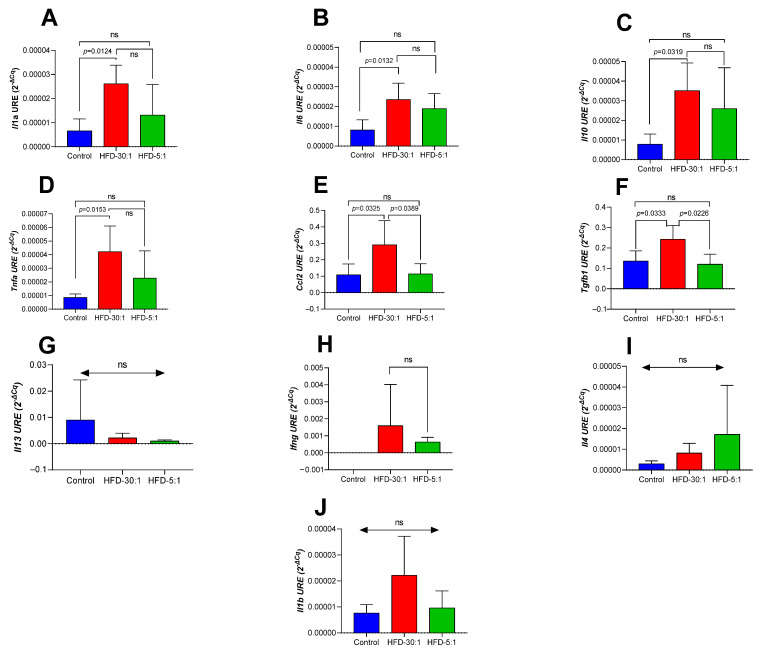
Expression levels of inflammation-related genes in adipose tissue following the 8-week dietary intervention. (**A**) Il1a expression; (**B**) Il6 expression; (**C**) Il10 expression; (**D**) Tnfa expression; (**E**) Ccl2 expression; (**F**) Tgfb1 expression; (**G**) Il13 expression; (**H**) Ifng expression; (**I**) Il4 expression; (**J**) Il1b expression. Values are presented as mean ± SD. ns: non-significant.

The expression assays highlighted distinct gene expression patterns between the control and HFD groups. Markers showing significant differences between the control and HFD-30:1 groups included *Il1a*, *Il6*, *Il10*, *Tnfa*, *Ccl2,* and *Tgfb1* (Figure 4A–F). In contrast, no significant differences were observed for *Il13*, *Ifng*, *Il4*, and *Il1b* between the control group and either of the obesity groups (HFD-30:1 or HFD-5:1) (Figure 4G–J).

Among the obesity groups, significant differences in *Ccl2* and *Tgfb1* expression were found between the HFD-30:1 and HFD-5:1 groups (Figure 4E,F). Additionally, *Ccl2* and *Tgfb1* expression levels in the HFD-5:1 group approached those observed in the control group, suggesting a modulation of these markers toward baseline levels under the modified dietary regimen (Figure 4E,F). Furthermore, *Ifng* expression was undetectable in the control group but was present in both HFD groups (HFD-30:1 and HFD-5:1), indicating a specific response to high-fat dietary conditions (Figure 4H).

#### 3.2.5. Antioxidant Gene Expression Profile

**Figure 5:**
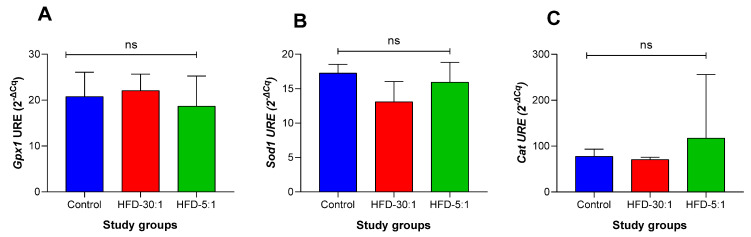
Effect of HFD-5:1 on antioxidant gene expression following the 8-week intervention. (**A**) Gpx1 expression; (**B**) Sod1 expression; (**C**) Cat expression. Values are presented as mean ± SD. ns: non-significant.

The expression analysis revealed no statistically significant differences in antioxidant gene expression (*Gpx1*, *Sod1*, and *Cat*) among the groups (Figure 5).

## 4. Discussion

Obesity is a major public health concern, affecting a significant proportion of the population and increasing the risk of type 2 diabetes mellitus, cardiovascular disease, and certain types of cancer [20]. In experimental research, diet-induced obesity models that simulate a Westernized dietary pattern in murine models are widely used to investigate mechanisms of weight gain and disruptions in insulin, lipid, and glucose metabolism [21,22]. In this study, a diet-induced obesity model was first established using two groups: a control and an HFD-30:1 group. After 17 weeks, the diet was modified to assess the effect of changing the polyunsaturated fatty acid (PUFA) ratio (*n*-6:*n*-3). This approach allowed us to directly evaluate the impact of reducing the dietary *n*-6:*n*-3 ratio on obesity-related metabolic and inflammatory outcomes.

The model successfully induced obesity, as evidenced by a 27% increase in body weight in the HFD-30:1 group compared to the control, along with a significantly higher epididymal adipose tissue mass, liver weight, and relative liver weight. These findings align with previous studies showing body and liver weight increases resulting from prolonged high-fat-diet consumption [23,24]. These anatomical changes emphasize not only the quantity but also the quality of dietary lipids, as an association was observed between lipid composition and tissue hypertrophy. This hypertrophy, in turn, contributes to disease development and multiple-organ dysfunction [25]. Importantly, the second phase of the experiment allowed us to explore the effects of changing the *n*-6:*n*-3 ratio from 30:1 to 5:1, isolating the role of lipid composition independent of caloric intake in modulating metabolic and inflammatory parameters.

In addition to the anatomical changes described above, significantly higher serum levels of triglycerides, cholesterol, and glucose were observed in the HFD-30:1 group compared to the control group at week 17. These metabolic alterations are typically associated with obesity and may be linked to adipose tissue expansion and dysfunction in response to the HFD-30:1. This dysfunction can promote the release of free fatty acids from adipocytes to the liver, leading to the formation of VLDL lipoproteins. In the murine model, this was reflected in elevated concentrations of cholesterol and triglycerides, as well as alterations in glucose homeostasis [21,26,27,28].

Moreover, this dietary pattern has been strongly associated with metabolic dysregulation through the promotion of an inflammatory environment related to insulin resistance. This occurs via interactions with the immune system, such as the release of chemokines and the recruitment of monocytes that polarize into classically activated M1 macrophages, along with the infiltration of granulocytes, lymphocytes, especially CD4+ Th1 cells and CD8+ T cells, and NK cells. All these events contribute to the establishment of low-grade inflammation [29,30,31].

In agreement, the first phase of this study highlighted changes in cytokine gene expression. In this sense, elevated expression levels of genes encoding both pro- and anti-inflammatory cytokines such as *Il1a*, *Il6*, *Il10*, *Tgfb1*, and *Il13* in the HFD-30:1 group compared with the control group emphasize the inflammatory state induced by the high-fat diet. This chronic inflammation is a well-known contributor to the pathogenesis of obesity and its complications [32].

Notably, the *Ifng* gene, which encodes another pro-inflammatory cytokine, was not expressed in the control group, suggesting that its expression is specifically induced by high-fat dietary conditions, particularly those rich in saturated fatty acids like palmitic acid. It has been reported that high-fat diets upregulate the expression of *Tlr2*, *Stat1*, and *Irf1*, which are transcription factors that enhance the expression of genes such as *Ifng*. Therefore, the observed expression of *Ifng* could be explained through this mechanism [33,34].

IFN-γ is known for its critical role in mediating inflammation and the activation of immune cells, particularly in the context of autoimmune diseases and infections [35,36]. In obesity, IFN-γ may contribute to the chronic inflammation characteristic of this condition, promoting insulin resistance and the development of metabolic comorbidities. IFN-γ stimulates macrophage polarization toward the M1 phenotype, which is pro-inflammatory and produces TNF-α, IL-6, and other cytokines. IFN-γ-induced inflammation contributes to insulin resistance by interfering with insulin signaling in adipocytes, hepatocytes, and skeletal muscle [37]. The specificity of *Ifng* expression under high-fat dietary conditions highlights the importance of an adequate dietary lipid intake to reduce its overexpression and suggests that IFN-γ may serve as a sensitive marker for diet-induced inflammation and as a potential therapeutic target.

Conversely, weight gain and dyslipidemia have been associated with a reduction in antioxidant enzyme activity. Obesity induced by a high-fat diet is accompanied by elevated oxidative stress in hepatic, cardiac, and renal tissues, characterized by a marked decrease in antioxidant enzyme activity [38]. Some studies have reported downregulation of *Sod1* and *Cat* genes in C57BL/6J male mice fed high-calorie diets, which increases oxidative stress in obesity [39]. In our study, no significant differences were observed in the expression of antioxidant enzymes (*Gpx1*, *Sod1*, *Cat*), consistent with findings reported by other authors [38]. However, other studies have reported such differences, possibly due to variations in the composition of the high-fat diet (HFD), particularly higher proportions of lard. This suggests that an increased intake of saturated fat sources may modulate hepatic antioxidant enzyme expression. These discrepancies may therefore be attributable to differences in dietary fat composition [40].

In the second phase of this study, HFD-fed mice were subdivided into two groups—HFD-30:1 and HFD-5:1—based on an 8-week dietary intervention aimed at modifying the *n*-6:*n*-3 PUFA ratio. A significant reduction in body weight was observed in the HFD-5:1 group compared to the HFD-30:1 group at the end of the 8-week period, which weights comparable to those of the control group. This underscores the importance of nutrient quality, as all groups had similar energy intakes, yet the HFD-5:1 group showed improved outcomes. Therefore, it is possible to achieve weight loss through a lipid-modified diet, supporting its potential relevance for translational research into dietary lipid modulation.

In addition to weight reduction, the HFD-5:1 group demonstrated improved biochemical parameters, including lower fasting glucose, triglyceride, and total cholesterol levels, which were comparable to those in the control group. These metabolic improvements were paralleled by enhanced insulin sensitivity, as evidenced by a reduced AUC in the ITT. This reflects better insulin sensitivity in the HFD-5:1 group compared to the HFD-30:1 group. Mechanistically, *n*-3 PUFA derivatives, particularly DHA, have been shown to activate GPR120, a G-protein-coupled receptor expressed in adipose tissue and macrophages. GPR120 activation enhances GLUT4 translocation and facilitates glucose uptake in adipocytes, contributing to improved insulin sensitivity [41].

Regarding triglyceride reduction, the proposed mechanisms include an inhibition of diacylglycerol acyltransferase (DGAT), the primary enzyme responsible for triglyceride synthesis in the liver. *N*-3 PUFAs also reduce de novo lipogenesis by inhibiting the transcription of the *SREBP1c* gene, which encodes a transcription factor that regulates genes involved in cholesterol, fatty acid, and triglyceride synthesis [42,43,44,45].

A previous study investigated the effects of *n*-6:*n*-3 ratios, such as 1:1 and 5:1 in T2DM models using plant-based *n*-3 sources (chia seed, palm, peanut, olive oil) over an 8-week period. The study found that a 5:1 ratio significantly reduced total cholesterol and fasting glucose by 23.84% and 20.6%, respectively (*p* < 0.05) [14].

In contrast, our study observed even greater reductions in total cholesterol (49.61%) and fasting glucose (28.20%) under the same 5:1 ratio. This may be due to the source of fatty acids: while the previous study used plant-based ALA-rich oils, our HFD-5:1 diet included menhaden oil, a marine-derived fat rich in eicosapentaenoic acid (EPA) and docosahexaenoic acid (DHA), unlike safflower oil, which primarily contains *n*-6 linoleic acid. EPA and DHA exhibit stronger lipid-lowering, anti-inflammatory, and insulin-sensitizing effects than ALA, due to their direct incorporation into membranes and modulation of genes involved in lipid metabolism [46].

In terms of cholesterol reduction, *n*-3 PUFA may decrease VLDL particle assembly in hepatocytes and reduce their secretion. This occurs via increased degradation of ApoB, the main protein necessary for VLDL assembly and secretion. Additionally, ALA promotes the conversion of cholesterol into bile acids through the action CYP7A1, enhancing cholesterol excretion [47,48].

Some human studies have shown that dietary cholesterol influences circulating LDL cholesterol levels. When relevant variables are controlled, modifying dietary cholesterol leads to significant increases in LDL. However, replacing saturated fats with unsaturated fats, especially polyunsaturated fats, consistently lowers total and LDL cholesterol and reduces cardiovascular risk [49]. In our study, the HFD-5:1 group consumed menhaden oil (rich in EPA/DHA), while the HFD-30:1 group received safflower oil (higher in saturated fat and *n*-6 PUFA). Interestingly, despite the HFD-5:1 group having a higher total dietary cholesterol content, their serum cholesterol was significantly lower. This underscores the importance of the fat source and type over the absolute cholesterol content in determining lipid metabolism and cardiovascular risk.

The greater metabolic benefits observed in the HFD-5:1 group likely reflect the higher EPA/DHA content of marine-derived *n*-3 sources. In contrast, plant-based *n*-3 (ALA) must be converted to EPA/DHA, and only 9% is converted in humans [50]. This highlights the differences in effectiveness between marine and seed oil-based PUFA sources.

Cytokines can be pro-inflammatory or anti-inflammatory. Pro-inflammatory cytokines (e.g., TNF-α, IL-1β, IL-6, MCP-1) contribute to metabolic disorders such as obesity and diabetes. Anti-inflammatory cytokines (e.g., TGF-β, IL-13) aim to counter this state, but may also play dual roles. Cytokines are secreted by a variety of immune cells, such as IL-1α from neutrophils and macrophages, and IL-10 from dendritic cells and NK cells. Immunomodulation of these molecules is a promising therapeutic strategy [51].

In this context, Van, R. et al. (2015) [32] reported higher expression of *Il1b*, *Il6*, and *Il10* in mice fed an HFD vs. low-fat diet for 24 weeks. Ji, Y. et al. (2012) also found increased *Il4* expression in adipose tissue from HFD-fed mice, possibly as a compensatory anti-inflammatory response [52].

In our study, significant differences in *Ccl2* and *Tgfb1* were observed between the HFD-30:1 and HFD-5:1 groups, with the HFD-5:1 group showing reduced levels, approaching those of the control group. This suggests a reduced pro-inflammatory state, highlighting the relevance of the PUFA composition in modulating immune responses in obesity. A favorable *n*-6:*n*-3 ratio (i.e., higher *n*-3 availability) may reduce the activation of inflammatory pathways and the release of pro-inflammatory metabolites [50]. It has also been reported that an HFD increases regulatory T cells to suppress adipose inflammation, potentially explaining the higher *Tgfb1* levels in the HFD group [53].

Additionally, immune cell infiltration into adipose tissue promotes the upregulation of MCP-1 and other pro-inflammatory cytokines. However, *n*-3 PUFA metabolites may suppress cytokine expression by inhibiting PKCα and PKCε, disrupting NF-kB translocation and mitigating inflammation through interruption in this signaling pathway [54].

In this sense, anti-inflammatory mechanisms of EPA and DHA include alterations in cell membrane phospholipid composition, disruption of lipid rafts, inhibition of the activation of pro-inflammatory transcription factors such as NFκB—thereby reducing the expression of inflammatory genes—and activation of the anti-inflammatory transcription factor peroxisome proliferator-activated receptor γ (PPARγ). These fatty acids are capable of partially inhibiting many aspects of inflammation, including leukocyte chemotaxis, adhesion molecule expression, leukocyte–endothelial adhesive interactions, and production of eicosanoids such as prostaglandins and leukotrienes derived from *n*-6 fatty acids. Moreover, EPA and DHA give rise to resolvins, and DHA also generates protectins and maresins, which are lipid mediators involved in the resolution of inflammation. Thus, the quantity, composition, and type of polyunsaturated fatty acids present in inflammatory cells significantly influence the function of these cells [55,56,57].

Regarding the effects of the HFD-5:1 diet on the expression of antioxidant enzyme genes, no statistically significant differences were observed among the groups. Although direct changes in gene expression were not detected, previous studies have shown that *n*-3 PUFAs can modulate transcription factors such as Nrf2, which regulates the expression of antioxidant enzymes and exerts cytoprotective effects against oxidative stress induced by hydrogen peroxide H_2_O_2_ [12]. Another study has reported changes in the enzymatic activity of antioxidant defenses following treatment with EPA and DHA [58]. These findings suggest that the antioxidant effects of *n*-3 PUFAs may occur through activation of the Nrf2 pathway or via post-transcriptional mechanisms.

Given the central role of Nrf2 in regulating the antioxidant response, it would be relevant for future research to analyze its expression and activation, including nuclear localization and potential post-translational modifications. Additionally, epigenetic or post-transcriptional regulatory mechanisms, such as promoter methylation of antioxidant genes or modulation by microRNAs, could influence gene translation without altering mRNA expression levels.

Furthermore, assessing oxidative stress markers, such as levels of reactive oxygen species (ROS), lipid peroxidation, or DNA damage, in future projects would help determine whether a high-fat diet disrupts redox homeostasis. This would offer a more comprehensive view of the antioxidant response in this experimental context.

Nevertheless, future studies are necessary to explore the potential immunomodulatory effects of varying *n*-6:*n*-3 PUFA ratios on cytokine signaling pathways in adipose tissue. Incorporating pathway-specific analyses and advanced cellular approaches such as single-cell transcriptomics, immune cell phenotyping, or in vitro co-culture models could provide deeper insights into the molecular mechanisms underlying the observed anti-inflammatory effects. These investigations could offer a clearer understanding of the translational potential of dietary lipid modulation as a strategy for managing obesity-related inflammation.

One of the limitations of this study is the relatively small sample size, which was selected based on the precedent from established experimental models. While the observed trends are biologically plausible and consistent with the previous literature, we recognize that not all effects can be unequivocally attributed to the modified *n*-6:*n*-3 PUFA ratio alone. Confounding variables, including the overall composition of dietary fats, the specific sources of fatty acids, and inter-individual metabolic variability, may have influenced the outcomes.

Therefore, future research should aim to further elucidate the complex mechanisms by which PUFA ratios modulate metabolic and inflammatory pathways. This includes the implementation of more robust experimental designs and larger sample sizes and the integration of multi-omics approaches to assess systemic and tissue-specific responses. Gaining a deeper understanding of these mechanisms will be essential for translating preclinical findings into effective, evidence-based dietary strategies for the prevention and treatment of obesity.

## 5. Conclusions

This study demonstrates that dietary modulation of the *n*-6:*n*-3 polyunsaturated fatty acid ratio from 30:1 to 5:1 in a murine model of diet-induced obesity leads to significant metabolic and inflammatory improvements. These findings underscore the importance of dietary lipid quality—rather than quantity alone—in shaping metabolic health and immune responses. Importantly, they support the potential of adjusting the *n*-6:*n*-3 ratio as a non-pharmacological, nutrition-based strategy to mitigate obesity-related low-grade inflammation and metabolic dysfunction. Future translational studies are warranted to explore its clinical applicability and to further elucidate the underlying molecular mechanisms in humans.

## Figures and Tables

**Table 1:** Composition of the control, HFD-30:1, and HFD-5:1 diets.

Components	Control		HFD-30:1		HFD-5:1	
	D12450H		D21022504		D21022505	
	g	% kcal	g	% kcal	g	% kcal
Proteins	19	20	24	20	24	20
Carbohydrates	67	70	41	35	41	35
Fat	4	10	24	45	24	45
Total		100		100		100
Kcal/g	3.8		4.7		4.7	
Ingredients	g	kcal	g	kcal	g	kcal
Casein	200	800	200	800	200	800
L-Cysteine	3	12	3	12	3	12
Cornstarch	452.2	1809	72.8	291	72.8	291
Maltodextrin 10	75	300	100	400	100	400
Sucrose	172.8	691	172.8	691	172.8	691
Cellulose	50	0	50	0	50	0
Lard	20	180	157	1413	157	1413
Menhaden oil	0	0	0	0	20.5	185
Safflower oil	0	0	45.5	410	0	0
Soy oil	25	225	0	0	25	225
Mineral mix S10026	10	0	10	0	10	0
Dicalcium phosphate	13	0	13	0	13	0
Calcium carbonate	5.5	0	5.5	0	5.5	0
Potassium citrate monohydrate	16.5	0	16.5	0	16.5	0
Vitamin mix	10	0	10	0	10	0
Choline bitartrate	2	0	2	0	2	0
Total	1055.05	4057	858.15	4057	858.15	4057
PUFA and cholesterol content						
*n*-6 (g/4057 kcal)	17.9		74.6		52.8	
*n*-3 (g/4057 kcal)	2.1		2.3		10.6	
EPA	0.0		0.0		2.9	
DHA	0.0		0.0		2.1	
*n*-6:*n*-3	8.4		30.2		5.0	
Linoleic acid (g/kg)	16.9		82.1		60.2	
Fat cholesterol (mg/4057 kcal)	14.4		117.4		233.3	
Total cholesterol (mg/4057 kcal)	14.4		233.4		233.3	

HFD: high-fat diet; kcal/g: kilocalories per gram; EPA: eicosapentaenoic acid; DHA: docosahexaenoic acid; *n*-6: omega-6; *n*-3: omega-3.

**Table 2:** Effect of the 8-week dietary intervention with HFD-5:1 on tissue weight.

Tissue	Control	HFD-30:1	HFD-5:1	*p*-Value
Epididymal adipose (g)	1.1 ± 0.4	1.3 ± 0.5	1.0 ± 0.4	0.4
Liver (g)	1.4 ± 0.1 ^a^	2.1 ± 0.4 ^b^	1.6 ± 0.4 ^a^	0.01
Relative liver weight	4.2 ± 0.6 ^a^	5.5 ± 1.4 ^b^	4.0 ± 0.2 ^a^	0.04

Values are presented as mean ± SD. Different superscript letters (a, b) indicate statistically significant differences among groups (*p* < 0.05). Relative liver weight to the ratio of liver weight to total body weight.

## Data Availability

The data supporting the findings of this study are available from the corresponding author upon reasonable request.

## Supplementary Materials

The following supporting information can be downloaded at https://www.mdpi.com/article/10.3390/nu17121996/s1, Table S1. TaqMan assay information for target genes; Figure S1: Body weight progression over the 17-week high-fat diet induction period; Table S2. Weights of epididymal adipose tissue and liver and relative liver weight at week 17; Figure S2. Serum levels of triglycerides, total cholesterol, and glucose at the end of the 17-week period; Figure S3. Expression profile of inflammatory cytokines in adipose tissue of control and HFD-30:1 groups at week 17; Figure S4. Expression profile of antioxidant enzymes in adipose tissue of control and HFD-30:1 groups at week 17.

## Abbreviations

ALA	Alpha-linolenic acid
ANOVA	Repeated measures analysis of variance
ApoB	Apolipoprotein B
AUC	Area under the curve
BMI	Body mass index
*Cat*	Catalase gene
*Ccl2*	Monocyte chemoattractant protein-1 gene
DHA	Docosahexaenoic acid
EPA	Eicosapentaenoic acid
GPR120	G-protein-coupled receptor 120
*Gpx 1*	Glutathione peroxidase 1 gene
HFD	High-fat diet
HFD-5:1	High-fat diet with *n*-6:*n*-3 ratio of 5:1
HFD-30:1	High-fat diet with *n*-6:*n*-3 ratio of 30:1
IFN-γ	Interferon gamma
*Ifng*	Interferon gamma gene
IL-1α	Interleukin-1 alpha
IL-1β	Interleukin-1 beta
IL-4	Interleukin-4
IL-6	Interleukin-6
IL-10	Interleukin-10
Il-13	Interleukin-13
ITT	Insulin tolerance test
MCP-1	Monocyte chemoattractant protein-1
*n*-3	Omega-3 fatty acids
*n*-6	Omega-6 fatty acids
NF-κB	Nuclear factor κappa B
PUFAs	Polyunsaturated fatty acids
ROS	Reactive oxygen species
SD	Standard deviation
*Sod 1*	Superoxide dismutase 1 gene
*Tgfb1*	Transforming growth factor beta 1 gene
TGF-β	Transforming growth factor beta
*Tnfa*	Tumor necrosis factor alpha gene
TNF-α	Tumor necrosis factor alpha
VLDL-c	Very-low-density lipoprotein cholesterol
WHO	World Health Organization
