# Supplementary material for: Dietary n-6:n-3 PUFA Ratio Modulates Inflammation-Related Gene Expression and Influences Improvements in Biochemical Parameters in a Murine Model of Diet-Induced Obesity
Source: Nutrients. 2025 Jun 13;17(12):1996. doi: 10.3390/nu17121996 (PMC12196131; doi:10.3390/nu17121996)
Supplement: Supplementary file 1 [file nutrients-17-01996-s001.zip › nutrients-3677517-supplementary.pdf]

Supplementary material

Table S1. Information of TaqMan probes used for the target genes.

Target genes	Assay ID
Il1a	Mm00439620_m1
Il1b	Mm00434228_m1
Il4	Mm00445259_m1
Il6	Mm00446190_m1
Il10	Mm00439616_m1
Il13	Mm00434204_m1
Tnfa	Mm00443258_m1
Ccl2	Mm00441242_m1
Tgfb1	Mm01178820_m1
Ifng	Mm01168134_m1
Gpx1	Mm00656767_m1
Sod1	Mm01344233_g1
Cat	Mm00437992_m1

Figure S1. Body weight progression over 17 weeks of intervention.

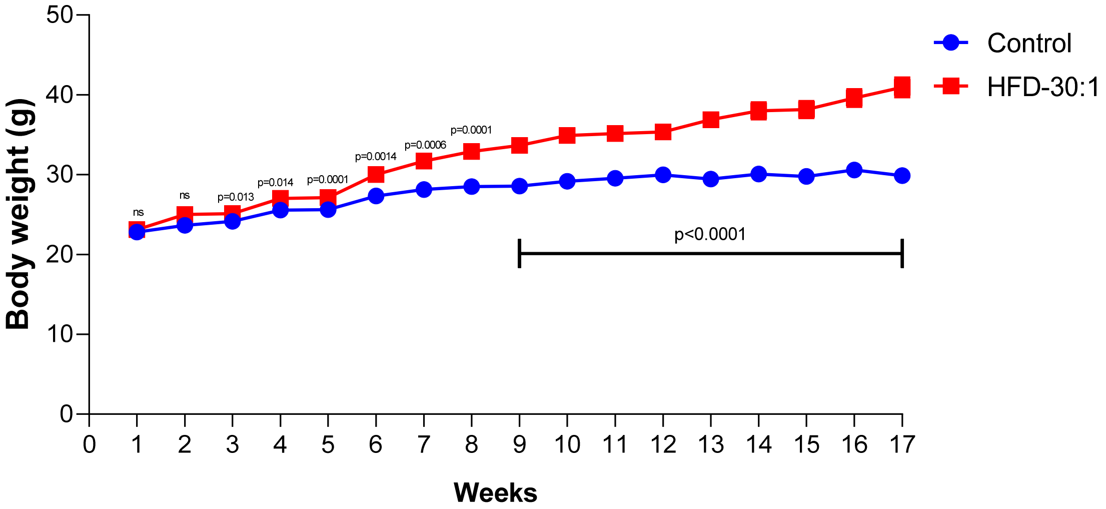

Values are presented as mean. ns = not significant.

Table S2. Epididymal adipose, liver weight, and relative liver weight at the final 17 weeks.

Tissue (gr)	Control	HFD-30:1	p-value
Epididymal adipose	0.63 ± 0.14	2.057 ± 0.86	0.001
Liver	1.136 ± 0.32	2.34 ± 0.88	0.005
Relative liver weight	3.72 ± 1.54	6.776 ± 2.31	0.039

Values are presented as mean ± SD. Relative liver weight is the ratio of liver weight to total body weight.

Figure S2. Levels of triglycerides, total cholesterol, and glucose at the final of 17 weeks

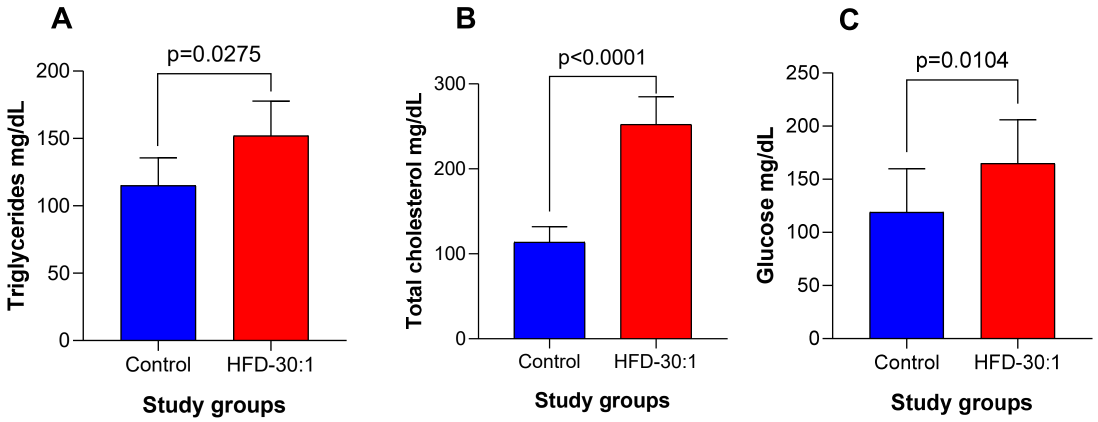

Values are presented as mean ± SD.

Figure S3. Cytokines expression profile in the control and HFD-30:1 groups at the end of 17 weeks.

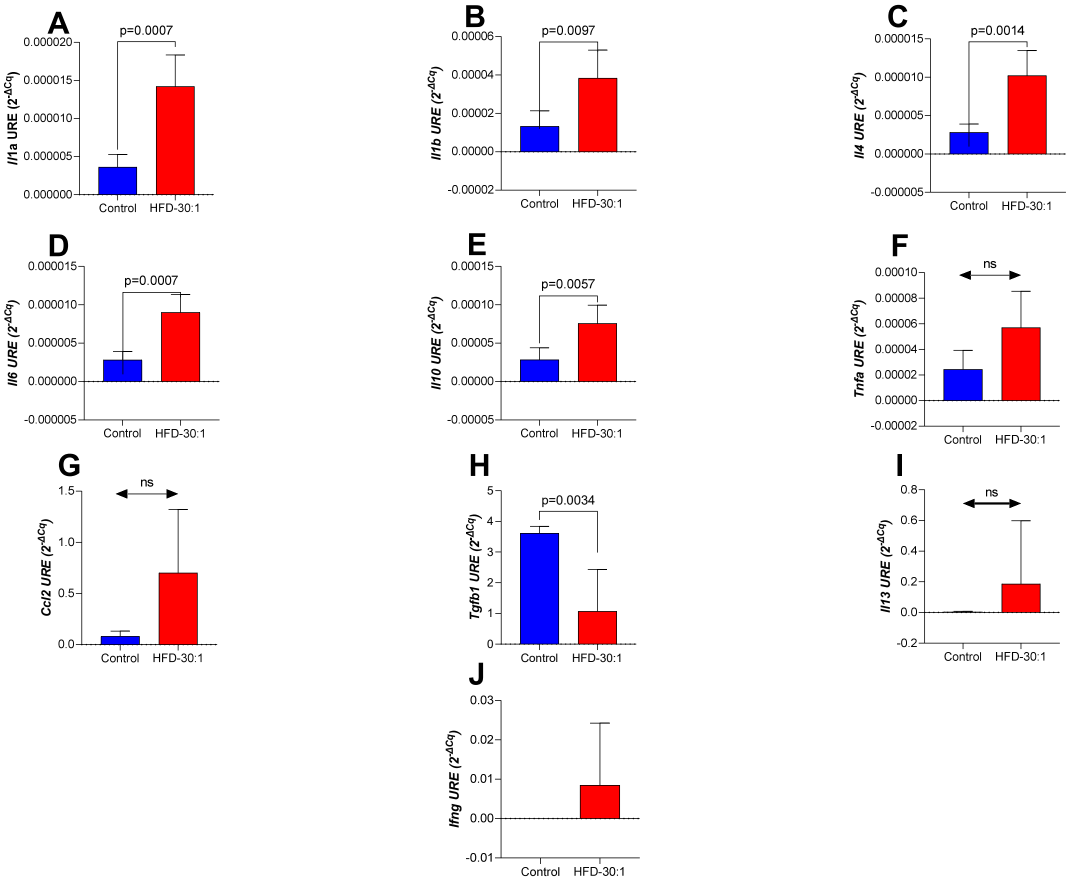

(A) *Il1a* expression, (B) *Il1b* expression, (C) *Il4* expression, (D) *Il6* expression, Panel E) *Il10* expression, (F) *Tnfa* expression, (G) *Ccl2* expression, (H) *Tgfb1* expression, (I) *Il13* expression, (J) *Ifng* expression. Values are presented as mean ± SD. Statistical significance is indicated as follows: ns = not significant.

Figure S4. Antioxidant enzymes expression profile in the control and HFD-30:1 groups at the end of 17 weeks.

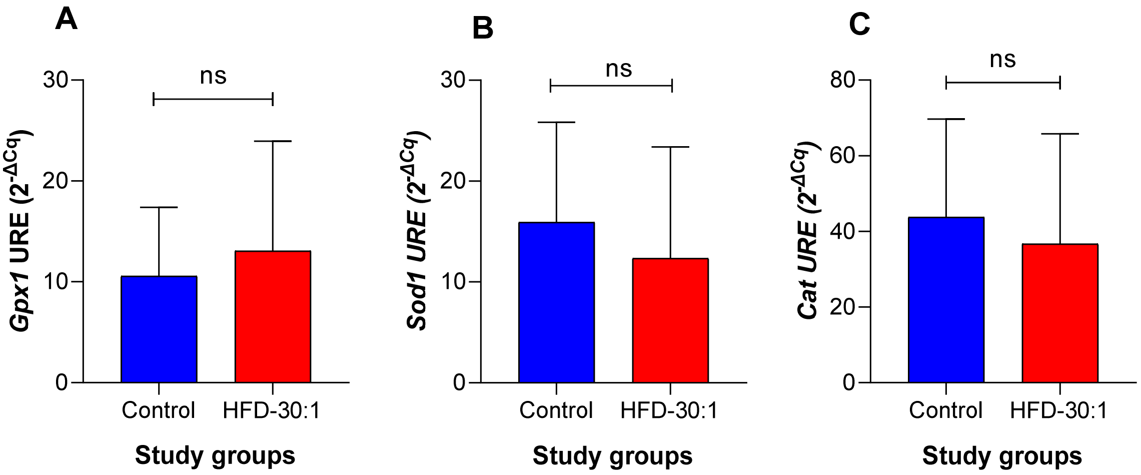

(A) *Gpx1* expression, (B) *Sod1* expression, and (C) *Cat expression*. Values are presented as mean ± SD. ns = not significant
